# VsMATE1-Mediated Citrate Efflux Is Involved in Al Resistance in Common Vetch (*Vicia sativa* L.)

**DOI:** 10.3390/plants14020290

**Published:** 2025-01-20

**Authors:** Wenhui Yan, Jia Shi, Ling He, Zigang Hou, Zhenfei Guo, Haifeng Zhu

**Affiliations:** College of Grassland Science, Nanjing Agricultural University, Nanjing 210095, China; 2022120019@stu.njau.edu.cn (W.Y.);

**Keywords:** aluminum resistance, common vetch, citrate efflux, *MATE1*

## Abstract

Planting aluminum-tolerant legume green manure is a cost-effective and sustainable method to increase soil fertility as well as decrease Al toxicity in acidic soils. By analyzing the relative root elongation of seven legume green manure species, common vetch (*Vicia sativa* L.) was identified as an Al-resistant species. Furthermore, cultivars 418 (cv. Sujian No.3) and 426 (cv. Lanjian No.3) were identified as Al-resistant and -sensitive cultivars, respectively, among 12 common vetch germplasms. The root growth of 418 was less inhibited by Al toxicity in both the germination stage and seedling stage than that of 426. Under Al toxicity, 418 accumulated less Al in both roots and shoots. Citrate is more abundant in the roots of common vetch compared to oxalate or malate. The internal citrate contents showed no significant difference between 418 and 426 under either control or Al treatment. However, the citrate efflux increased in response to Al in 418 but not in 426 and was higher in 418 under Al stress than in 426. Consistently, *VsMATE1* expression increased faster and to a greater extent in 418 than 426 in response to Al stress. These results indicated that a VsMATE1-mediated citrate efflux might play an important role in Al resistance in common vetch. It is suggested that *VsMATE1* is a valuable candidate gene for aluminum resistance breeding.

## 1. Introduction

Al toxicity is a serious problem for crop production in acid soils. It inhibits root elongation and shoot growth and leads to decreased crop yield and quality. Green manure application is a valuable strategy to improve acidic soils by increasing soil fertility and pH and decreasing Al toxicity. The incorporation of green manures significantly increases the soil pH and microbial biomass carbon compared with fallow treatments [1]. It may enhance crop production by increasing nitrogen and phosphate availability as well as reducing exchangeable Al concentrations in acidic soil [2]. Fresh green manure (*Chamaecrista rotundifolia* cv. Wynn) shows remarkable effects on Al detoxification shortly after application, which is associated with the release of low molecular organic acids during the decomposition of green manure [3]. Therefore, it is important to identify Al-tolerant green manure species or varieties and study the molecular mechanisms of Al tolerance for the further application of green manure in acidic soils.

Leguminous plants are widely planted as green manure or cover crops on farms due to their ability to fix atmospheric N into organic forms through biological nitrogen fixation. Many Al-tolerant legume species have been identified and used in acidic soils. Evaluation of soil acidity tolerance in 14 tropical legume cover crops via a greenhouse experiment revealed that Jack bean, black Mucuna, and gray Mucuna bean species were the most tolerant varieties, while Brazilian lucerne and tropical kudzu were the most susceptible [4]. Among fourteen white clover (*Trifolium repens* L.) cultivars from eleven countries, “Grasslands Huia” white clover has the highest Al tolerance [5]. *Adesmia latifolia* has the most plastic root in acidic soil compared to *Trifolium repens* or *Trifolium pratense* [6]. *Lupinus* spp is used as a break crop in the semi-arid region in Western Australia for its high tolerance to acidic soils [7,8]. Stylo (*Stylosanthes guianensis*) exhibits superior Al tolerance and is one of the most important tropical legumes used in a wide range of agricultural systems with acidic soils [9]. Common vetch (*Vicia sativa* L.) is an annual leguminous crop and widely planted as green manure and forage [10,11]. Common vetch is successfully recommended under drought conditions for its high tolerance to drought [12]. In a previous study, common vetch performed much better than *Medicago sativa* and *Astragalus sinicus* in soils with pH 5.65 [13]. Common vetch was also recommended in acid citrus orchard soils to control weeds and improve the soil quality [14]. To broaden its use in acidic soils, it is important to identify Al-resistant common vetch germplasms. However, the Al tolerance of common vetch and the underlying mechanisms has not been reported.

To cope with Al toxicity, plants have evolved two main types of strategies: Al exclusion that aims to prevent Al from entering the roots and Al tolerance that is achieved by Al detoxification and sequestering. The most well-characterized Al exclusion mechanism is Al-dependent root exudation of organic acid (OA) anions into the rhizosphere, where they chelate Al^3+^ ions to form nontoxic compounds. OA exclusion mechanism have been elucidated in many legumes. Al toxicity increases citrate release, and LaALMT1 mediates malate exudation in Lupin [15,16,17]. Malate secretion mediated by *SgALMT2* contributes to the ability of stylo to cope with Al toxicity [9]. Both citrate and malate were induced by Al toxicity in soybean, and citrate was noted as the main OA exudate [18]. The MATE proteins within citrate transport functions have been identified from many leguminous plants, such as *Medicago sativa*, soybean, and chickpea [19,20,21,22,23,24]. The MATE family was identified as hub genes in the co-expression network of Al response in elephant grass roots, and overexpression of *CpMATE93* conferred Al resistance in yeast cells [20]. Five MATE proteins, GmMATE13, GmMATE47, GmMATE75, GmMATE79, and GmMATE87, are involved in Al-induced citrate secretion from soybean [21,22]. Overexpressing *GmMATE13* in soybean hairy roots enhanced Al resistance by increasing citrate efflux [23]. Sugarcane plants constitutively overexpressing the *Sorghum bicolor MATE* gene (*SbMATE*) showed improved tolerance to Al [24]. However, *MATE* genes in common vetch have not been studied.

In this study, we identified common vetch as an Al-resistant legume. Furthermore, Al-tolerant common vetch germplasms were identified for potential use in breeding, and Al tolerance mechanisms in common vetch was studied.

## 2. Results

### 2.1. Growth Response to Al in Seven Legume Species 

**Figure 1:**
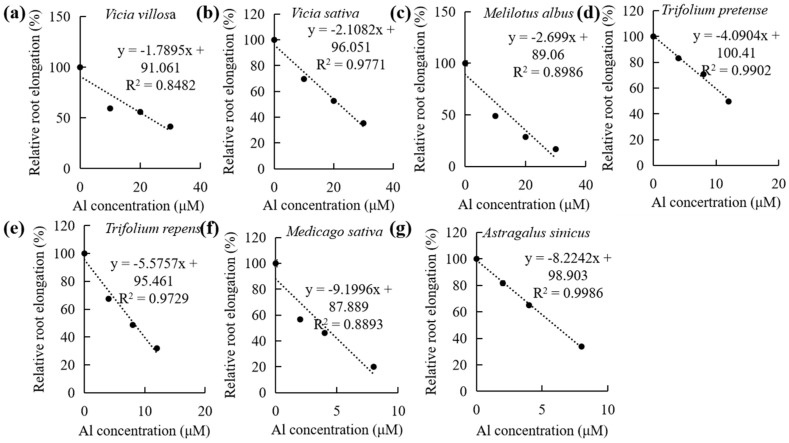
Correlation between relative root elongation and Al concentration in *Vicia villosa* (**a**), *Vica sativa* (**b**), *Melitotus albus* (**c**), *Trifolium pretense* (**d**), *Trifolium repens* (**e**), *Medicago sativa* (**f**), and *Astragalus sinicus* (**g**). Seedlings were treated with a 0.5 mM CaCl_2_ solution (pH 4.5) containing different concentrations of AlCl_3_ for 24 h. Root length was measured, and the relative root elongation was calculated. The correlation between Al concentration and relative root elongation was obtained via linear regression analysis, and semi-inhibitory concentrations were calculated.

Relative root elongation of seven legumes were measured. Four different Al concentration gradient were set: 0, 10, 20, and 30 µM for *Vicia villosa*, common vetch, and *Melitotus albus*; 0, 4, 8, and 12 µM for *Trifolium pretense* and *Trifolium repens*; and 0, 2, 4, and 8 µM for *Medicago sativa* and *Astragalus sinicus* (Figure S1). As Al concentration increased, the relative root elongation decreased, and a good correlation between Al concentration and relative root elongation was obtained in all legumes with an R^2^ higher than 0.84 (Figure 1). Common vetch and *Vicia villosa* are Al-resistant legumes with semi-inhibitory concentrations at about 22 µM. *Medicago sativa* is super sensitive to Al with a semi-inhibitory concentration at about 4 µM.

### 2.2. Comparison of Al Tolerance in Eleven Common Vetch Collections

**Figure 2:**
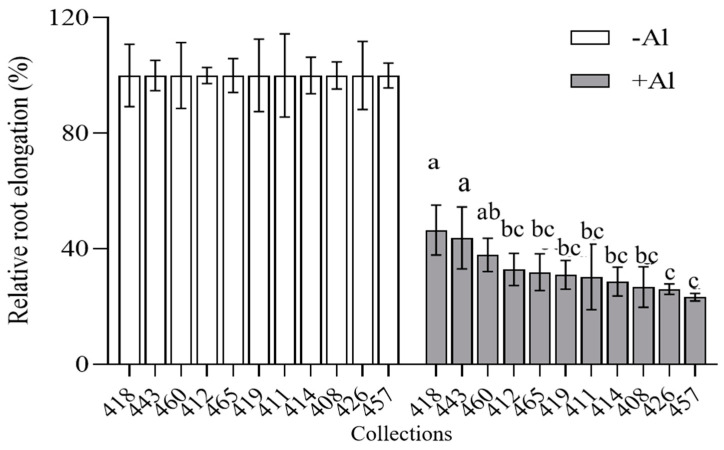
Analysis of Al tolerance in common vetch collections. The germinated seeds were treated with 0.5 mM CaCl_2_ solution (pH 4.5) containing 0 or 25 µM AlCl_3_ for 24 h. Root length was measured, and the relative root elongation was calculated. Data are means ± SD (n = 10). Columns with different letters indicate significant differences at *p* < 0.05 (one-way ANOVA followed by Tukey’s post hoc test).

The germinated seeds in eleven common vetch collections were exposed to 0 or 25 µm AlCl_3_ for 24 h for measurement of relative root elongation. Cultivars 415 and 418 had higher relative root elongations of 56% and 46%, respectively, while 426 and 457 had the lowest relative root elongations of 28% each among the tested collections (Figure 2). The results indicated that 415 and 418 were Al-resistant and 426 was Al-sensitive. The Al-resistant 418 and Al-sensitive 426 were used for the subsequent research.

### 2.3. The Al-Resistant Common Vetch Accumulated Less Al than the Al-Sensitive One

**Figure 3:**
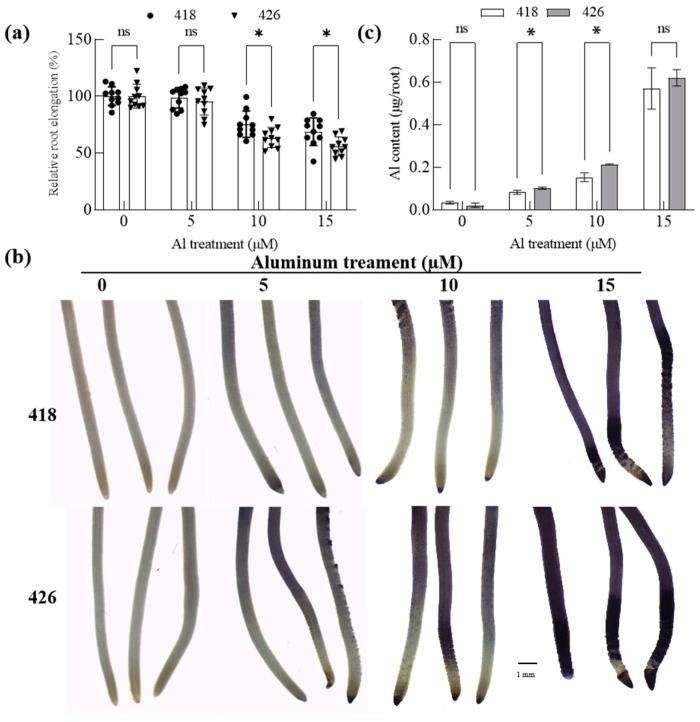
Effect of Al toxicity on relative root elongation and Al accumulation in roots of Al-resistant cultivar (418) and Al-sensitive cultivar (426) at germination stage. Five-day-old seedlings were treated with 0.5 mM CaCl_2_ solution (pH 4.5) containing 0, 5, 10, or 15 µM AlCl_3_ for 24 h. (**a**) Root length was measured, and the relative root elongation was calculated. Data are means ± SD (n = 10). n.s. and * represent no and significant differences at *p* < 0.05 (two-way ANOVA followed by Tukey’s post hoc test; multiple comparisons were made between 418 and 426). (**b**) Roots were stained by hematoxylin and photographed. Bar = 1 mm. (**c**) Aluminum in root surface was extracted by soaking roots (0–2 cm) in 1 N nitric acid for more than 2 days, and Al content was analyzed by ICP-OES. Data are means ± SD (n = 3). n.s. and * represent no and significant differences at *p* < 0.05 (two-way ANOVA followed by Tukey’s post hoc test; multiple comparison was made between 418 and 426).

**Figure 4:**
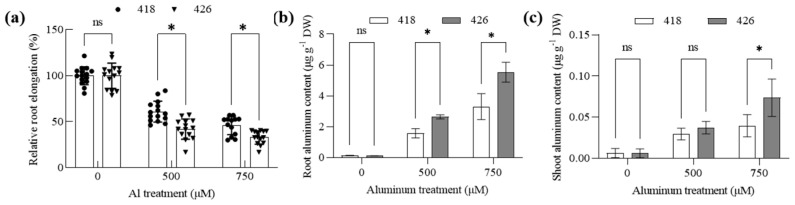
Effect of aluminum on root elongation and Al content in root of Al-tolerant cultivar (418) and Al-sensitive cultivar (426) in seedling stage. Two-week-old seedlings were treated with 1/2 Hoagland solution (pH 5.0) containing 0, 500, or 750 µM AlCl_3_ for 12 days. (**a**) Root length was measured, and the relative root elongation was calculated. Data are means ± SD (n = 15). (**b**,**c**) Al content in roots and shoots were analyzed. Data are means ± SD (n = 3). n.s. and * represents no and significant differences at *p* < 0.05 (two-way ANOVA followed by Tukey’s post hoc test; multiple comparisons were made between 418 and 426).

Seedlings of 418 and 426 were treated with 0, 5, 10, and 15 µm AlCl_3_ for 24 h. Subsequently, roots were either stained with the Al indicator dye hematoxylin or harvested for analysis of Al accumulation via inductively coupled plasma mass spectrometry (ICP-OES). Root elongation of 418 was less inhibited than that of 426 after treatment with 10 and 15 µm AlCl_3_ (Figure 3a). The roots in 426 displayed a more intense staining under Al conditions compared to 418 (Figure 3b). Consistently, ICP-OES-dependent quantification of Al accumulation revealed that more Al was accumulated in root tips (0–2 cm) in 426 after treatment with 5 and 10 µm AlCl_3_ compared to 418 (Figure 3c). Furthermore, ten-day-old seedlings were exposed to one-half Hoagland nutrient solution (pH 5.0) containing 0, 500, or 750 mM AlCl_3_ for ten days. Root growth was less inhibited with lower Al accumulation in roots and shoots than 426 under Al conditions (Figure 4). The results indicated that an Al exclusion mechanism might play an important role in Al resistance in 418.

### 2.4. The Al-Resistant Common Vetch Released More Citrate than the Al-Sensitive One

**Figure 5:**
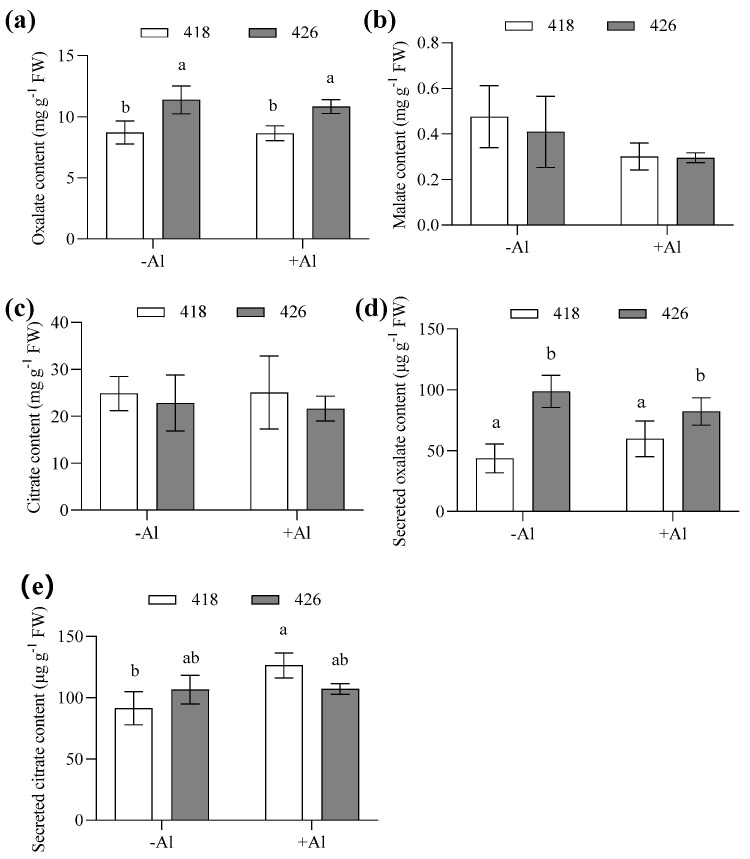
Effect of aluminum on internal root organic acid and secreted citrate content on Al-resistant cultivar (418) and Al-sensitive cultivar (426). Two-week-old seedlings were treated with 0.5 mM CaCl_2_ solution (pH 4.5) containing 0 or 15 µM AlCl_3_ for 24 h. (**a**) Oxalate, (**b**) malate, and (**c**) citrate contents in roots; (**d**) secreted oxalate; and (**e**) citrate content in cultivation solution were measured using HPLC. Data are means ± SD (n = 4). Columns with different letters indicate significant differences at *p* < 0.05 (two-way ANOVA followed by Tukey’s post hoc test; multiple comparisons were made between columns).

**Figure 6:**
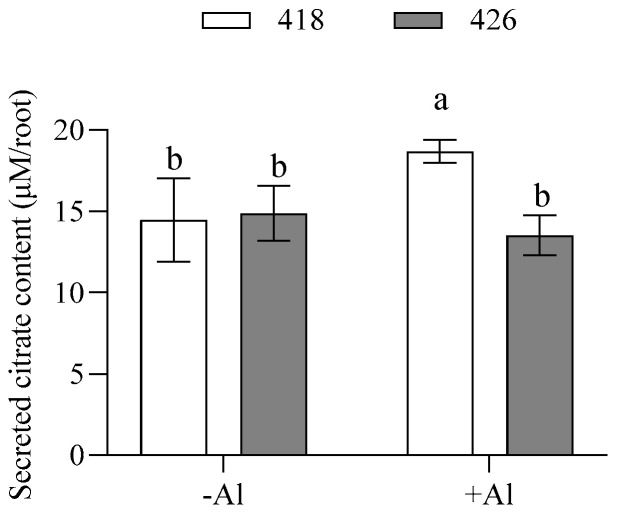
Citrate efflux from roots of Al-resistant cultivar (418) and Al-sensitive cultivar (426) in response to Al toxicity. Five-day-old seedlings were treated with 0.5 mM CaCl_2_ solution (pH 4.5) containing 0 or 15 µM AlCl_3_ for 12 h. Secreted citrate contents in cultivation solution were measured with an enzyme assay as described previously [25]. Data are means ± SD (n = 4). Columns with different letters indicate significant differences at *p* < 0.05 (two-way ANOVA followed by Tukey’s post hoc test; multiple comparisons were made between columns).

Internal and external OA content was quantified via high-performance liquid chromatography (HPLC) in cultivars 418 and 426 in response to Al. There were no significant differences in the internal malate and citrate contents between 418 and 426 in either the −Al or +Al groups, while internal oxalate content in 426 was slightly higher than 418 (Figure 5a–c). Cultivar 426 also secreted more oxalate than 418 in both the −Al and +Al groups, while no significant differences were detected between the -Al and +Al groups, indicating that oxalate secretion was not induced by Al stress in common vetch (Figure 5d). Secreted malate was barely detected by the HPLC method. On the other hand, there were no significant differences in the citrate secretion between 418 and 426 under −Al condition. However, 418 secreted more citrate than 426 after 24 h of Al treatment (Figure 5e). To confirm this, the enzymatic method was used to detect citrate secretion in response to Al. It was revealed that 418 secreted more citrate than 426 after 12 hours of Al treatment (Figure 6).

### 2.5. VsMATE1 Responds to Al More Dramatically and Faster in the Al-Resistant Common Vetch than the Sensitive One

**Figure 7:**
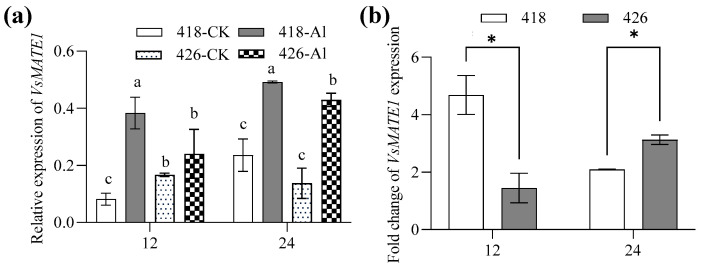
Relative expression of VsMATE1 in Al-resistant cultivar (418) and Al-sensitive cultivar (426). Five-day-old seedlings were treated with 0.5 mM CaCl_2_ solution (pH 4.5) containing 0 or 15 µM AlCl_3_ for 24 h. (**a**) Relative expression of *VsMATE1* in roots was analyzed by qRT-PCR. (**b**) The relative expression level under Al stress was divided by the relative expression level in the control group to get the fold change of *VsMATE1* expression. Data are means ± SD (n = 3). Columns with different letters indicate significant difference at *p* < 0.05 (two-way ANOVA followed by Tukey’s post hoc test; multiple comparisons were made between columns). n.s. and * represents no and significant differences at *p* < 0.05 (two-way ANOVA followed by Tukey’s post hoc test; multiple comparisons were made between 418 and 426).

*VsMATE1* was identified and cloned from common vetch; it showed 63.32% identity with *AtMATE* in an ammonia acid sequence (Figure S2). Quantitative real-time PCR was used to detect *VsMATE1* expression in 418 and 426. *VsMATE1* transcript was increased in response to Al stress in both 418 and 426. *VsMATE1* was upregulated by three times in 418 after 12 h Al treatment, while it was not significantly upregulated in 426 until 24 h after Al treatment (Figure 7).

## 3. Discussion

### 3.1. Common Vetch Is an Al-Resistant Legume with Great Potential for Application in Acidic Soils

Excessive Al in acidic soils is a primary factor that hinders plant growth. Some Al-resistant legumes, such as Lupin, Stylo, and *Chamaecrista rotundifolia,* have been used in acidic soils in rotation or via an intercropping agricultural system. To expand the potential application of legumes in acidic soils, we evaluated the Al resistance of seven legumes. Our work revealed that common vetch and *Vicia villosa* are Al-resistant legumes at a semi-inhibitory concentration of about 22 µM in 0.5 mM CaCl_2_ solution (pH 4.5), which was much higher than those in *Melitotus albus*, *Trifolium pretense*, *Trifolium repens*, *Medicago sativa*, and *Astragalus sinicus*. However, about 4 µM AlCl_3_ can inhibit root elongation by half in *Medicago sativa*, indicating its extreme Al sensitivity, consistent with previous reports [26].

Previous work indicated that common vetch can grow well in acidic soils (pH 5.65) with a dry matter yield of 2.78 t/ha, higher than alfalfa or milk vetch [13]. To identify Al-resistant common vetch germplasms for future application in acidic soils, Al resistance of eleven germplasms were evaluated. It was revealed that Al resistance differs between germplasms. Germplasms 415 (cv. Xinjiang jianwan) and 418 (cv. Sujian No.3) were relatively resistant to Al toxicity, while 457 and 426 (cv. Lanjian No.3) were Al-sensitive. Phosphate deficiency is also a major restrictive factor for crop production in acidic soils, and plants have developed the ability to utilize OA to combat both Al toxicity and *p* deficiency [27,28]. Therefore, it raises an open question of whether Al-resistant plants are more resistant to Pi deficiency. Our previous work revealed that 418 and 426 are phosphate deficiency-tolerant cultivars, while 415 is sensitive to phosphate deficiency [29]. It seems that there are no significant correlations between Al resistance and phosphate deficiency tolerance, consistent with Karim’s work in wheat [30]. This indicates that 418 (Sujian No.3) might be valuable for use as a forage or cover crop in farmland or orchards, as reported in [13,14].

### 3.2. VsMATE1-Mediated Citrate Exclusion May Play an Important Role in Al Resistance in Common Vetch

Plants employ various mechanisms to counter Al toxicity, including Al exclusion from the roots by exuding organic acids, the ability to tolerate Al in the symplast, or both. Citrate, oxalate, and malate have been identified as the major OA anions secreted by plant roots exposed to Al stress. All three of these anions are able to chelate Al, but their chelating abilities differ, following the order citrate > oxalate > malate [31]. Therefore, citrate, oxalate, malate, and Al contents in Al-resistant 418 and Al-sensitive 426 were compared to study the Al resistance mechanisms in common vetch. Al-exclusion mechanisms appeared to explain Al resistance in 418, with less Al accumulation in roots and shoots and more citrate exclusion than 426. There were no significant differences in the internal malate and citrate contents between 418 and 426 in either the -Al or +Al groups, while oxalate content in 426 was slightly higher than in 418. Under Al stress, citrate secretion in 426 was significantly increased, while oxalate secretion was not changed in either cultivar, indicating that citrate secretion may play a vital role in Al detoxification in common vetch, consistent with Lu’s work [32]. Increased amounts of organic acids not only regulate Al^3+^ concentrations but also enhance phosphate uptake and shape the microbial community composition at the root–soil interface [33]. Previous studies have investigated the structure of rhizosphere bacterial communities in Al-tolerant (Al-T, secreted more OAs) and Al-sensitive soybean cultivars and suggested that Al-T genotypes recruit certain bacterial species that help mitigating Al toxicity [34]. It would be interesting to investigate the rhizosphere microbial communities in 418 and 426 in the future.

The multidrug and toxic compound extrusion (MATE) family of OA/H^+^ antiport transporters is responsible for plasma membrane citrate efflux [35,36]. We determined the *AtMATE* homologous gene *VsMATE1* in common vetch by searching in our RNA-seq database and reported the genome sequence [37]. Expression of *VsMATE1* was upregulated after 12 hours’ Al treatment in 418 but not 426, and the *VsMATE1* transcript level in 418 was higher than that in 426 under Al toxicity, consistent with the OA secretion. These results indicate that VsMATE1 might mediate the citrate efflux under Al stress. Numerous *MATE* genes involved in citrate efflux have been identified in plant species, which has enabled investigation of the transcript-level regulation of these genes in response to Al stress. These studies revealed that these transporter genes’ expression is usually higher in Al-resistant genotypes than in Al-sensitive genotypes [38], similar to our results. All these observations suggest that regulation of the expression of genes encoding transporter proteins enables adaptation to Al toxicity conditions. *VsMATE1* expression is expected to be a selection marker for Al-resistant common vetch; therefore, our work facilitates the future design of gene-specific markers for Al-tolerant line selection in common vetch breeding programs.

## 4. Materials and Methods

### 4.1. Plant Materials, Growth Conditions, and Treatments

Seven legume species, *Vicia sativa* cv. Sujian No.3, *Vicia villosa* cv. Jianghuai, *Trifolium pratense* cv. Amos, *Trifolium repens* cv. Haifa, *Medicago sativa* cv. Algonquin, *Astragahuis sinicus* cv. Yijiangzi, and *Melilotus officinalis*, were used. Eleven common vetch collections were used, as listed in Table S1. Seeds were sterilized using NaClO and kept at 4 °C for 3 days before germinating at room temperature. The germinated seeds were transferred to nets floating on a 0.5 mM CaCl_2_ solution in a 4 L plastic container in a growth chamber with 16 h light/8 h dark, 24 °C/20 °C, and 65% relative humidity. For Al tolerance evaluation at the germination stage, germinated seeds with 1–3 cm roots were pretreated with a 0.5 mM CaCl_2_ solution at pH 4.5 for 24 h before being exposed to a 0.5 mM CaCl_2_ solution containing different concentrations of AlCl_3_ at pH 4.5 for 24 h. For Al tolerance evaluation at the seedling stage, seedlings with one true leaf were placed in a modified one-half Hoagland nutrient solution (pH 5.0) containing 0, 500, or 750 mM AlCl_3_. The one-half Hoagland nutrient solution contained the following macronutrients (in mM)—Ca, 2.5; K, 3.0; Mg, 0.5; PO_4_^−^, 0.5; NO^3−^, 8.0; NH_4_^+^, 0.5; and SO_4_^2−^, 0.5—and micronutrients (in µM)—Fe-EDTA, 20; Mn, 9.0; Zn, 0.76; Cu, 0.32; MoO_4_^−^, 0.12; H_3_BO_3_, 20; and Cl^−^, 18. pH was adjusted with a 1 N HCl solution. The nutrient solution was renewed every two days. The relative root elongation was calculated by dividing root elongations by the mean elongation of the control group.

### 4.2. Determination of Al Content

The seedlings were treated with 0, 5, 10, or 15 µM AlCl_3_ (0.5 mM CaCl_2_, pH 4.5) for 24 h. For hematoxylin staining, roots were washed three times with 0.5 mM CaCl_2_ at pH 4.5 and then stained with hematoxylin for 30 min. The dyed roots were washed in sterile water for 30 min and subsequently observed and imaged through a somatotype microscope (SZX16, OLYMPUS, Tokyo, Japan). To analyze nitric-acid-extracted Al content, root tips (0–2 cm) with three biological replicates were sampled from seedlings under both –Al and +Al conditions. Ten root segments were collected in each sample and soaked in 1 mL nitric acid (1 N) for 2 d. The Al content in the extraction solution was determined using inductively coupled plasma mass spectrometry (ICP-OES, Optima 8000, PerkinElmer, Waltham, MA, USA). For determination of Al concentrations in roots and shoots at the seedling stage, seedlings were exposed to one-half Hoagland nutrient solution (pH 5.0) containing 0, 500, or 750 mM AlCl_3_ for ten days. After been washed in sterile water, roots and shoots were dried at 60 °C in an oven for a week and digested with nitric acid. The Al concentration was measured by ICP-OES.

### 4.3. Measurements of Internal and External OAs

Organic acids were measured using high-performance liquid chromatography (HPLC, UltiMate 3000, Thermo, Waltham, MA, USA) and the enzymatic method. For the HPLC method, two-week-old seedlings were treated with a 0.5 mM CaCl_2_ solution (pH 4.5) containing 0 or 15 µM AlCl_3_ for 24 h. Internal and external citrate, oxalate, and malate contents were measured as reported in [19]. HPLC parameters were as follows: chromatographic column, ACQUITY UPLC HSS T3 (100 mm × 2.1 mm × 1.8 µm, Waters, Milford, MA, USA); mobile phase, 20 mM KH_2_PO_4_ in 1% methanol buffer (pH 2.7); flow rate, 0.25 mL min^−1^; UV wavelength, 214 nm; injection volume, 20 µL; and running time, 15 min. All samples were subjected to a 0.45 µm membrane suction filtration. Four independent biological replicates were performed. For enzymatic method, every three two-day-old seedlings were treated with 2 mL 0.5 mM CaCl_2_ (pH 4.5) containing 0 or 15 µM AlCl_3_ for 12 h with four biological replicates. Citrate content in the solution was determined as reported in [25].

### 4.4. Identification and Cloning of VsMATE1

*VsMATE1* was identified by BLASTP with an E-value cutoff at 1e-5 from a database (NCBI accession number PRJNA1102286) using the ammonia sequence of *AtMATE* from *Arabidopsis*. After searching in SMART (http://smart.embl-heidelberg.de/smart/set_mode.cgi?NORMAL=1 (accessed on 6 November 2023)), *VsMATE1* gene that contained the completed core domain of each family were selected. DNA sequence of *VsMATE1* was obtained from the common vetch genome [37] and confirmed by cloning and sequencing.

### 4.5. RNA Extraction and Quantitative Real-Time PCR (qRT-PCR)

Total RNA was extracted by the RNAprep pure Plant Kit (Tiangen Inc., Beijing, China). cDNA was synthesized using the HiScript III RT SuperMix for qPCR Kit (Vazyme, Nanjing, China). The diluted cDNA was used as a template according to the ChamQ SYBR qPCR Master Mix (Vazyme, Nanjing, China) instructions in the Thermal Cycler Dice™ Real-Time System (Takara, Otsu, Japan). The Unigene 68614 was used as an internal reference gene [39]. The forward and reverse primers for *VsMATE1* were 5’-AGACAGTCTTGGTCGGGAGA-3’ and 5’-TATTTGGCCAATGAATGCA-3’. The relative expression level was calculated based on 2^−ΔΔCt^. The fold change of relative expression was calculated by dividing relative expressions by the relative expression of the control group.

## 5. Conclusions

In conclusion, common vetch is more resistant to Al than *Trifolium pratense*, *Trifolium repens*, *Medicago sativa*, *Astragahuis sinicus*, and *Melilotus officinalis*. In eleven common vetch collections, cultivars 415 (cv. Xinjiang jianwan) and 418 (cv. Sujian No.3) ranked high for Al resistance. By comparing the different responses of Al-resistant and Al-sensitive cultivars to Al toxicity, it was suggested that citrate secretion may play a vital role in Al detoxification in common vetch. Furthermore, *VsMATE1* was induced more rapidly and to a greater extent by Al stress in the Al-resistant cultivar compared to the Al-sensitive one, suggesting that *VsMATE1* may paly a vital role in citrate efflux in response to Al toxicity.

## Data Availability

The data that support the findings of this study are available from the corresponding author upon reasonable request.

## Supplementary Materials

The following supporting information can be downloaded at: https://www.mdpi.com/article/10.3390/plants14020290/s1, Figure S1: Aluminum tolerance of *Vicia villosa*, *Vica sativa*, *Melitotus officinalis*, *Trifolium pratense*, *Trifolium repens*, *Medicago sativa*, and *Astragalus sinicus*; Figure S2: Alignment of VsMATE1 and AtMATE sequence; Table S1: Germplasm resources of common vetch (*Vicia sativa* L.).

## Abbreviations

The following abbreviations are used in this manuscript:

Al	Aluminum
OA	Organic acid
ICP-OES	Inductively coupled plasma mass spectrometry
HPLC	High-performance liquid chromatography
