# Supplementary material for: VsMATE1-Mediated Citrate Efflux Is Involved in Al Resistance in Common Vetch (Vicia sativa L.)
Source: Plants (Basel). 2025 Jan 20;14(2):290. doi: 10.3390/plants14020290 (PMC11769015; doi:10.3390/plants14020290)
Supplement: Supplementary file 1 [file plants-14-00290-s001.zip › supplement.pdf]

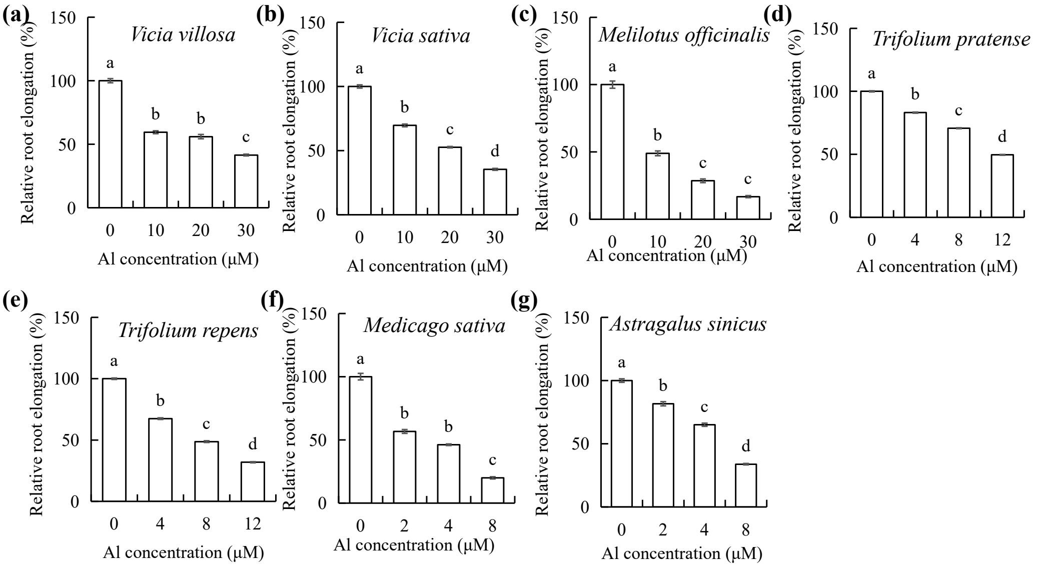

Figure S1 Aluminum tolerance of *Vicia villosa* (a), *Vica sativa* (b), *Melitotus officinalis* (c), *Trifolium pratense* (d), *Trifolium repens* (e), *Medicago sativa* (f) and *Astragalus sinicus* (g). Seedlings were treated with 0.5 mM $CaCl_2$ solution (pH 4.5) containing different concentration of $AlCl_3$ for 24 h, root length was measured and the relative root elongation was calculated. Data are means ± SE (n = 10). Columns with different letters indicate significant difference at $P < 0.05$ (One-way ANOVA followed by Turkey's post hoc test).

```
VsMATE1   MNQKESVFSSTSDWMRIPVLTFFKDARLVFKSDSLGREILSIALPAAMALTADPIASLVDTAFIGQIGPVELAAVGVSIA   80
AtMATE    MMSED...GYNTDFPRNPLYIFFSDFRSVLKFDELGLEIARIALPAALALTADPIASLVDTAFIGQIGPVELAAVGVSIA   77
Consensus m           d  r p   ff d r v k d lg ei  ialpaa altadpiaslvdtafigqigpvelaavgvsia

VsMATE1   LFNQASRIAIFPLVSVTTSFVAEEDTLSGANSQVEEN.GCLEAATP.LDAETKEFLPQKNLD..TESFN......LVKNV  150
AtMATE    LFNQVSRIAIFPLVSITTSFVAEEDACSSQQDTVRDHKECIEIGINNPTEETIELIPEKHKDSLSDEFKTSSSIFSISKP  157
Consensus lfnq sriaifplvs ttsfvaeed  s     v     c e        et e  p k  d     f

VsMATE1   EHKRRHIPSASSALFIGGILGIIQAILLISAAKPLLSFMGVTSDSPMLHPAQQYLKLRSLGAPAVLLSLAMQGVFRGFKD  230
AtMATE    PAKKRNIPSASSALIIGGVLGLFQAVFLISAAKPLLSFMGVKHDSPMMRPSQRYLSLRSLGAPAVLLSLAAQGVFRGFKD  237
Consensus   k r ipsassal igg lg  qa  lisaakpllsfmgv  dspm  p q yl lrslgapavllsla qgvfrgfkd

VsMATE1   TKTPLYATVAGDATNIALDPLFIFVFRMGVTGAAIAHVISQYLISAILLWSLKKQVDLIPPSMKHLQFDRFLKNGFLLLM  310
AtMATE    TTTPLFATVIGDVTNIILDPIFIFVFRLGVTGAATAHVISQYLMCGILLWKLMGQVDIFNMSTKHLQFCRFMKNGFLLLM  317
Consensus t tpl atv gd tni ldp fifvfr gvtgaa ahvisqyl   illw l  qvd    s khlqf rf kngflllm

VsMATE1   RVIAVTFCVTLAASLAARHGSTSMAAFQVCLQVWLAVSLLADGLAVAGQAILAGAFANKDYEKATATASRVLQMGLVLGA  390
AtMATE    RVIAVTFCVTLSASLAAREGSTSMAAFQVCLQVWLATSLLADGYAVAGQAILASAFAKKDYKRAAATASRVLQLGLVLGF  397
Consensus rviavtfcvtl aslaar gstsmaafqvclqvwla slladg avagqaila afa kdy  a atasrvlq glvlg

VsMATE1   ALAFILGTGLHFGAKIFTKDANVLHLIRIGIPFVALTQPLNSLAFVFDGVNFGASDFAYSAFSMVCIFFLHVLVHTYLIE  470
AtMATE    VLAVILGAGLHFGARVFTKDDKVLHLISIGLPFVAGTQPINALAFVFDGVNFGASDFGYAAASLVMVAIVSILCLLFLSS  477
Consensus  la ilg glhfga  ftkd  vlhli ig pfva tqp n lafvfdgvnfgasdf y a s v       l    l

VsMATE1   PLKIT................................                                              475
AtMATE    THGFIGLWFGLTIYMSLRAAVGFWRIGTGTGPWSFLR                                              514
Consensus
```

Figure S2 Alignment of VsMATE1 and AtMATE sequence.
